# Novel insights into abdominal wall hernia (AWH) and its negative impact on patients’ finances: “Doing my job was pretty impossible”

**DOI:** 10.1007/s10029-026-03596-9

**Published:** 2026-02-04

**Authors:** Olivia Smith, Asim Abbas, Mark Mierzwinski, Andrew Bertram, Praminthra Chitsabesan, Srinivas Chintapatla

**Affiliations:** 1 York Abdominal Wall Unit, Dept. of General Surgery, York and Scarborough Teaching Hospitals NHSFT, York, YO31 8HE UK; 2 School of Science, Technology and Health, York St. John University, York, UK; 3 Department of Finance, York and Scarborough Teaching Hospitals NHSFT, York, YO31 8HE UK

## Background

Unemployment is strongly correlated with ill health [1]. Health reasons are one reason people can become unemployed [2–4]. Indeed, people with pathology are more likely to be unemployed [5] or absent from work for prolonged periods [6]. Furthermore, job loss, or the feeling of not being able to perform one’s job, is associated with financial disruption, loss of assets, social withdrawal [7], and family disturbance. Such disturbance can often contribute to declines in psychological [8] and physical well-being [9, 10], which not only diminish life satisfaction but can serve to prolong unemployment, perpetuating a vicious cycle. The importance of contextual factors, such as societal norms regarding work and gender expectations, which influence the severity of unemployment’s impact on psychological outcomes has also been shown [11].

Complex abdominal wall hernia (CAWH) is increasingly recognised as a condition with wide-ranging consequences extending beyond physical symptoms. While research has explored pain [12], body image [13], sexual dysfunction [14], functional restriction and mental health [15] in this population, the socioeconomic dimensions remain largely underexamined. Despite evidenced links between health and unemployment, there is a paucity of literature examining the effect of CAWH on employment. No qualitative study to date has explored in depth how CAWH shapes patients’ employment trajectories, financial security, occupational identity, and perceived role within the family unit. Understanding the experiences of CAWH patients is particularly relevant with respect to Quality of Life, as their significant physical limitations and surgical recovery can disrupt employment, potentially creating cycles of financial and psychological strain. Therefore, this study is the first to qualitatively examine CAWH patients’ experience of employment and financial disturbance. This study seeks to address this gap by using a phenomenological methodology to examine how individuals living with CAWH understand and experience the financial and employment-related consequences of their condition. By foregrounding patients’ narratives, this work aims to illuminate neglected aspects of disease burden and inform more holistic preoperative counselling, postoperative support, and broader models of care.

## Methods

### Study design

This study adopted a phenomenological approach, qualitatively exploring human issues and subjects which can be emotionally laden. This approach suited our research focus and aim, to examine a patient’s lived experience of CAWH. Therefore, we used Interpretative Phenomenological Analysis (IPA) to examine participants’ lived experiences of the financial and employment disturbances CAWH can cause [16].

### Ethics

This research received study approval from the Hull York Medical School (HYMS), Integrated Research Approval System (IRAS) and Health Research Authority (HRA). It was conducted in accordance with the Declaration of Helsinki and has been reported according to COnsolidated criteria for REporting Qualitative (COREQ) guidelines [17]. The study protocol, patient information leaflet, consent forms, topic guide and interview schedules were designed and subject to an iterative approval process. All patients provided written and verbal informed consent.

### Recruitment

Participants were identified from the CAWH clinic at the York Abdominal Wall Unit. The sample was purposive, allowing for variation amongst participants, and included patients of all Ventral Hernia Working Group (VHWG) grades, those with previous cancers, previous wound infection, stoma, intestinal fistula, COPD, diabetes, smokers and obesity. A letter of invitation, along with an information sheet pertaining to the study, was sent to participants by the York Abdominal Wall Unit research team.

Purposive sampling was used to capture a broad range of lived experiences across age, sex, employment status, comorbidity burden, and surgical stage. This approach aligns with phenomenological methodology, which seeks depth and diversity of personal meaning rather than statistical representativeness. CAWH is a heterogeneous condition encountered across working-age and older adults, and our sampling strategy was designed to reflect the demographic and clinical variation present in routine clinical practice. Selection sought to capture a breadth of lived experiences and disease severity rather than statistical representativeness, consistent with phenomenological methodology. The inclusion of preoperative and postoperative patients reflected the natural distribution of clinic attendance during the study period rather than an a priori decision to construct two comparison groups. The intention was not to contrast pre- and postoperative cohorts, but to ensure representation of both experiences within the overall phenomenon of living with CAWH.

### Data collection

Semi-structured interviews were conducted by a Surgical Research Fellow (OS), using a topic guide, conducted between January and July 2020. The topic guide and interview schedule were tested in a small pilot study and refined (Supplementary File 1 and 2). Questions relating to finance and employment were contained within the topic guide and facilitated semi-structured discussion of these topics. For participants in paid employment, the semi-structured topic guide also included prompts relating to workplace functioning (e.g., ability to meet physical or cognitive demands), sickness absence, relationships with colleagues or employers, job security, and any adjustments or role changes necessitated by the hernia. For participants who were retired or not engaged in paid employment, interview questions were adapted to explore the impact of CAWH on functional independence, ability to undertake household tasks, caregiving responsibilities, social participation, and perceived role within the family unit. All participants were asked to reflect on their current lived experience rather than recollections from earlier life stages, ensuring that accounts centred on the contemporary impact of CAWH.

Three interviews were face to face interviews and the rest were telephone interviews due to the Covid-19 pandemic. Each interview lasted between 45 and 90 min, and was recorded by dictaphone. Steps were taken to ensure trust worthiness of process by asking open questions, clarity check of answers, using prompts and probes. The interview recording was transcribed verbatim by a medical secretary independent of the research team. Pseudonyms were used to ensure anonymity and confidentiality. OS engaged in reflexivity and field notes were made throughout the data collection process.

### Analysis

Data analysis was an iterative process until thematic saturation. Participant transcripts were analyzed using IPA within NVivo v12 (https://www.qsinternational.com/nvivo/home*).* Transcripts were analysed line by line to identify codes that may be grouped into a certain theme. Emergent themes were discussed with two gastrointestinal surgeons and two plastic surgeons who specialized in CAWR as well as an independent academic qualitative researcher, who does not have a surgical background (MM). This allowed triangulation of the findings as well as plausibility of results.

## Results

**Fig. 1:**
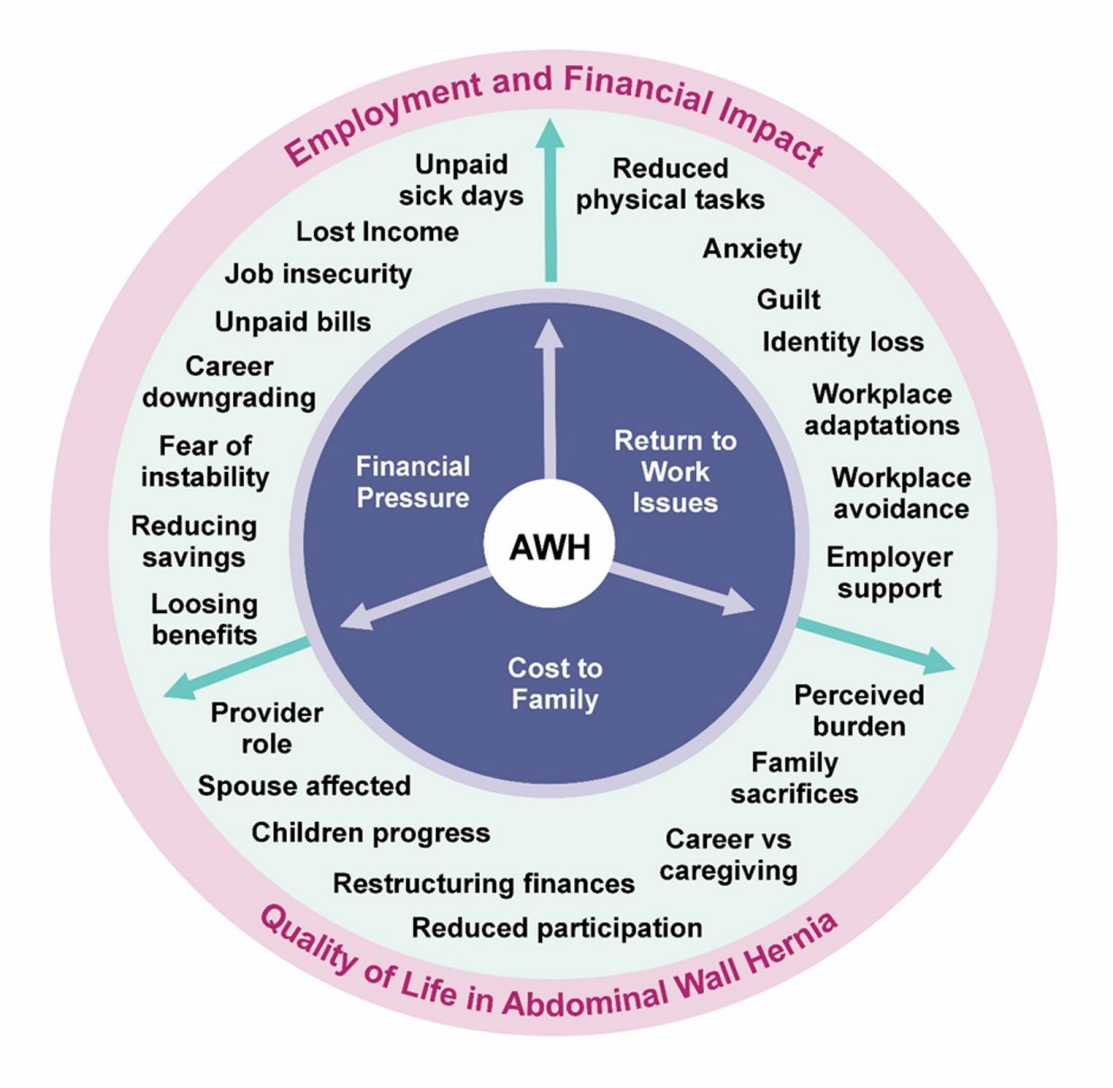
Employment and Financial impact of AWH

Participants included 8 men and 7 women aged between 36 and 85 years (median = 65 years) covering all VHWG grades (Supplementary File 3 details their biographies). Participant social, biological, and clinical characteristics (with pseudonymised names) are summarised in Table 1. "Employment and Finance" and its relevance to participants emerged as a theme on phenomenological interpretation of the transcripts. Three key themes pertaining to ‘employment and finance’ were identified: ‘financial pressure’, ‘return to work issues’ and ‘costs to family’; together visually represented in Fig. 1.

**Table 1:** Note: This data is mandatory. Please provide.

Participant	Age	Sex	Employment status	BMI	Key comorbidities	VHWG grade	Operative status
Agnes	65	F	Employed	29.6	Stoma	2	Pre-op
Betty	63	F	Retired	26.1	None	1	Pre-op
Charlotte	68	F	Retired	38.6	Diabetes, wound infection	4	Pre-op
David	61	M	Employed	31.2	Diabetes	2	Pre-op
Eric	78	M	Retired	29.9	None	1	Pre-op
Frank	75	M	Retired	25.8	Diabetes, stoma, cancer	4	Post-op
George	45	M	Employed	30.5	Diabetes, wound infection	4	Pre-op
Harry	84	M	Employed	28.8	Diabetes, stoma	2	Post-op
Ian	58	M	Employed	30.2	Diabetes, stoma	4	Pre-op
Joan	75	F	Retired	26.3	Stoma, wound infection	3	Pre-op
Kevin	74	M	Retired	32.4	Wound infection	3	Post-op
Lisa	39	F	Employed	29.2	None	1	Post-op
Marge	36	F	Employed	20.4	None	1	Post-op
Norman	77	M	Retired	24.1	Diabetes, stoma	3	Post-op
Ophelia	44	F	Employed	30.7	None	1	Post-op

### Theme 1: financial pressure

Financial pressure emerged as a significant theme concerning employment. Many participants described challenges such as unpaid sick days, hospital visits and, in some circumstances, job loss. These financial pressures contributed to, and compounded, the psychological toll of navigating employment disruptions and changes to personal identity.

George, a labourer and digger driver, shared his experience of eventually losing a job due to frequent sick days caused by his hernia. He described his symptoms of pain and increased bowel frequency as being impractical for his work environment. This situation profoundly impacted his mental health and motivation, with him stating,

*“I’ve lost a lot of interest in work. Work is hard at the moment.”*

George’s condition also created a sense of vulnerability and panic, especially regarding access to toilets whilst at work:

*“It has affected my work a lot as well, because I worry now if there’s going to be a toilet where I’m working. With being a digger driver, I could be in the middle of anywhere…we dig a lot of driveways out, and if the customer isn’t in (to use their toilet), I panic…I panic now going to work. I should maybe change jobs. I don’t know, I don’t know what to do.”*

Although George expressed a desire to seek alternative employment, he took pride in being good at his current job when able and acknowledged the significant financial repercussions of changing jobs.

*“I think I’m at the top of my game with digger driving, if I change and go somewhere else I’m at the bottom of the ladder and money won’t be as good. I won’t enjoy it. … I’m stuck.”*

A loss of finances and fiscal pressure were experiences also shared by Lisa and Marge. However, these experiences could contrast between those self-employed and those entitled to “sick pay”. Lisa, for example, noted whilst experiencing decreased income,

*“I’m quite lucky in that sense that as a teacher and I’m not self-employed, so I do have time allowed for sick pay.”*

These narratives highlight the dual burden of financial strain and identity disruption that many patients experienced. While George and Lisa provide vivid examples of employment-related disruption, variations of financial pressure were reported across the cohort. For some (particularly older or retired participants) the burden related to increased dependence on family, travel costs to hospital appointments, or managing household expenses on fixed incomes. Thus, although manifesting differently, financial strain was a recurrent concern expressed by the majority of participants.

### Theme 2: return to work issues

Participants reported two forms of struggle in the workplace; difficulties undertaking the required physical tasks and subsequent feelings of *“inadequacy”* and *“letting teammates down”.* As such, those employed in more physically intensive jobs disproportionately reported struggling in the workplace. This narrative was particularly true for Harry, Lisa and Marge who work in fitness, and became burdened by pain and restricted movements. Again, such struggles left participants feeling increasingly vulnerable, highlighted by Marge, a 36 year old woman who works with young offenders as well as a part-time personal trainer:

“*I work with offenders, I felt a little bit more vulnerable than I would generally have done… if they kicked off I felt more vulnerable. Normally…I’m the sort of person where I would go straight in and try and sort out the problem, but…I’d step back a bit and I was a bit more cautious because I thought well, it would be harder if they come to attack me. I just felt vulnerable. I just thought it (the hernia and pain related to it) could go at any time.”*

Having given up fitness classes because of her hernia, it was important for Marge to retain her identity in the workplace even though it led to feelings of loneliness, alienation and inadequacy in the workplace.

George expressed frustration, feelings of failure and guilt for *“letting his team down”*,* explaining*:

*“We do a lot of demolitions and I can’t go on demolitions now and pick*,* you know*,* pick pieces of wood out or anything. My boss understands…it’s just frustrating that I can’t do anything like that anymore”.*

Expressing similar concerns, Lisa stated:

*“I’m a PE teacher actually doing my job was pretty impossible. I wasn’t able to even physically get into a position to be able to support students safely. In terms of my job I just had to stop doing certain things… I had to stop actually teaching certain things and I really adapted the way I taught*,* which is not ideal really*,* when it is quite a physical job.”*

*“it made me feel was incredibly guilty for the team that I was working in*,* I felt like I was letting them down. I hate taking time off work*,* because then they have to cover or someone has to come in and teach the groups and*,* the groups of children didn’t get the stability (of teachers)”.*

Demonstrating a shift from their norms, possible momentary identity disruption and the subsequent feelings associated with this induced shift, Lisa, Harry, and Marge appeared to experience inner conflict, questioning their abilities to perform their job functions, and therefore their role as a financial provider within their family units.

The experiences shared by George and Lisa illustrate this theme, but similar concerns were articulated by participants across a range of occupations. For example, participants working in fitness, manual labour, and public-facing roles described guilt, frustration, and perceived inadequacy when unable to meet workplace expectations. These narratives were consistent across pre- and postoperative participants and across age groups.

### Theme 3: cost to family

Participants’ concern and negative experiences often centred on threats to their role as a provider within their family units that their CAWH had initiated. This was true for males and females, regardless of age. Whilst most had not experienced job loss before and were in “professional” careers such as teaching, those experiencing financial difficulties were non-discriminate in terms of gender and age. Initially not ready to accept changes in employment, participants, like George, confessed to finding it difficult to taper their negative emotions associated with their employment issues and changes, largely due to the removal of their financial independence.

Reflecting on their capacity to seek new perspectives related to their position as a financial provider, Lisa, Marge, and Harry turned to problem-focused strategies. For example, referring to potentially changing career even though it felt like a compromise and personal sacrifice, Lisa quoted:

*“I started to seriously contemplate well maybe I shouldn’t be a PE teacher, maybe I should start thinking about teaching another subject.”*

Furthermore, whilst Lisa stopped being a skiing instructor and personal trainer as she struggled to undertake these tasks, she retrained as a yoga instructor, strengthening her psychological and physical health, whilst providing necessary additional income to regain her sense of purpose and role as a provider. Lisa’s transition from skiing instruction and PE teaching to yoga instruction reflected her search for a role that allowed greater control of pace, reduced physical strain, and the ability to adapt movements around her hernia. Although yoga is a physical discipline, she described it as less demanding on the abdominal wall and more compatible with her symptoms than her previous roles.

Other participants cultivated different strategies such as helping others through volunteering and Norman considered downsizing his house to lower expenditure. To move forward, some participants demonstrated openness to new ways of thinking and to new situations to help with their role as a provider. For participants above retirement age, the impact of CAWH manifested less through employment loss and more through reduced functional autonomy, difficulty performing household or caregiving roles, and increased reliance on partners or family members. Several older participants described discomfort, reduced mobility, and fear of sudden hernia-related symptoms as limiting their independence in daily activities. Although financial strain was less often linked to income loss in this group, indirect costs - such as reliance on others, travel expenses, and reduced ability to engage in valued activities - were frequently described as sources of frustration and emotional burden. 

## Discussion

CAWH patients described job loss, job insecurity, and how the subsequent financial burden and stress had a detrimental psychological impact. This impact was disproportionately experienced by self-employed participants, especially those with manual jobs or for those who worked in the fitness industry. Such disruption was largely caused by pain and reduced physical ability, and the long recovery to pre-operative levels of physical condition. As such, many participants detailed losses in occupational identity, contributing to poorer self-esteem, which was further compounded by fears or actual experiences of no longer being able to effectively financially provide for their family. Postoperative reflections from several participants suggest that surgical repair may partially restore occupational identity and reduce financial stress, although this recovery is not universal. Some individuals continued to experience limitations or the residual consequences of prolonged absence from work. These findings underscore that postoperative support may be necessary even after successful repair.

Whilst our small sample size did not show significant cross-sectional variance, when scaled it is not inconceivable to consider disproportionate gender and social class inequalities. Despite continued inequities in many areas, in the 21 st century, England has witnessed substantial attitudinal and actual shifts away from traditional divisions of labour. However, men are significantly disproportionately more likely to have manual labour jobs and are more likely to be self-employed [18, 19]. In a post-industrial English service-based economy many working class people still have manual labour jobs, and they still view their physical prowess as one of their few attainable sources of power [20].

It is difficult to discuss our key findings in specific relation to literature concerning CAWH given its sparsity in this respect. Inguinal and ventral hernia have been associated with work-related risk factors such as load lifted per day [18, 21], but there is little literature pertaining to the number of days or jobs lost because of this disease. Our findings over anecdotal evidence that CAWH can contribute to a significant number of days lost from work. We can also consider how participants’ disruption to and, sometimes, loss of occupational identity resonates with latent functions of employment theory [19]. In this sense, employment not only provided financial stability but also offered a sense of purpose, time structure, and social connectivity, all of which were disrupted for participants. Understandably, this loss was pronounced amongst participants who strongly identified with their professions, such as teachers or fitness instructors, and those in manual labour roles that emphasized physical prowess.

The impact of CAWH varied according to life stage. For participants of working age, employment disruption, sickness absence, and income instability were dominant concerns. In contrast, for participants in later life or retirement, the consequences of CAWH were more commonly expressed through loss of functional autonomy, reduced ability to undertake household or caregiving roles, and increased reliance on partners or family members. Although not framed as ‘job loss’, these experiences were nonetheless associated with psychological distress and indirect financial strain.

The findings also highlight the broader economic and personal burden associated with CAWH, reinforcing the importance of prevention through evidence-based abdominal wall closure techniques and perioperative optimisation. High-quality fascial closure (‘small bites’), prophylactic mesh in high-risk patients, and robust postoperative pathways may reduce the incidence of complex hernias and their associated direct, indirect and intangible costs.

### Pragmatic solutions

Our key findings raise significant questions concerning quality of life and have informed changes to our patient care package. It is essential to refine and adapt CAWH Quality of Life assessment tools to capture the broader financial, occupational, and psychosocial impacts. Acknowledging the potential impact of CAWH, healthcare providers should integrate pre- and post-operative counselling involving employment and mental health. Occupational health specialists could help patients anticipate and adapt to workplace challenges. More broadly, multidisciplinary care teams should provide psychological support and resilience training, helping patients manage the stress and identity disruptions associated with employment loss. Additionally, clinicians could advocate for or provide access to vocational rehabilitation services, including retraining programs and job-matching initiatives tailored to the physical limitations imposed by CAWH. Finally, promoting employer education around CAWH and advocating for workplace accommodations could reduce stigma and facilitate a smoother return to work for patients.

**Fig. 2:**
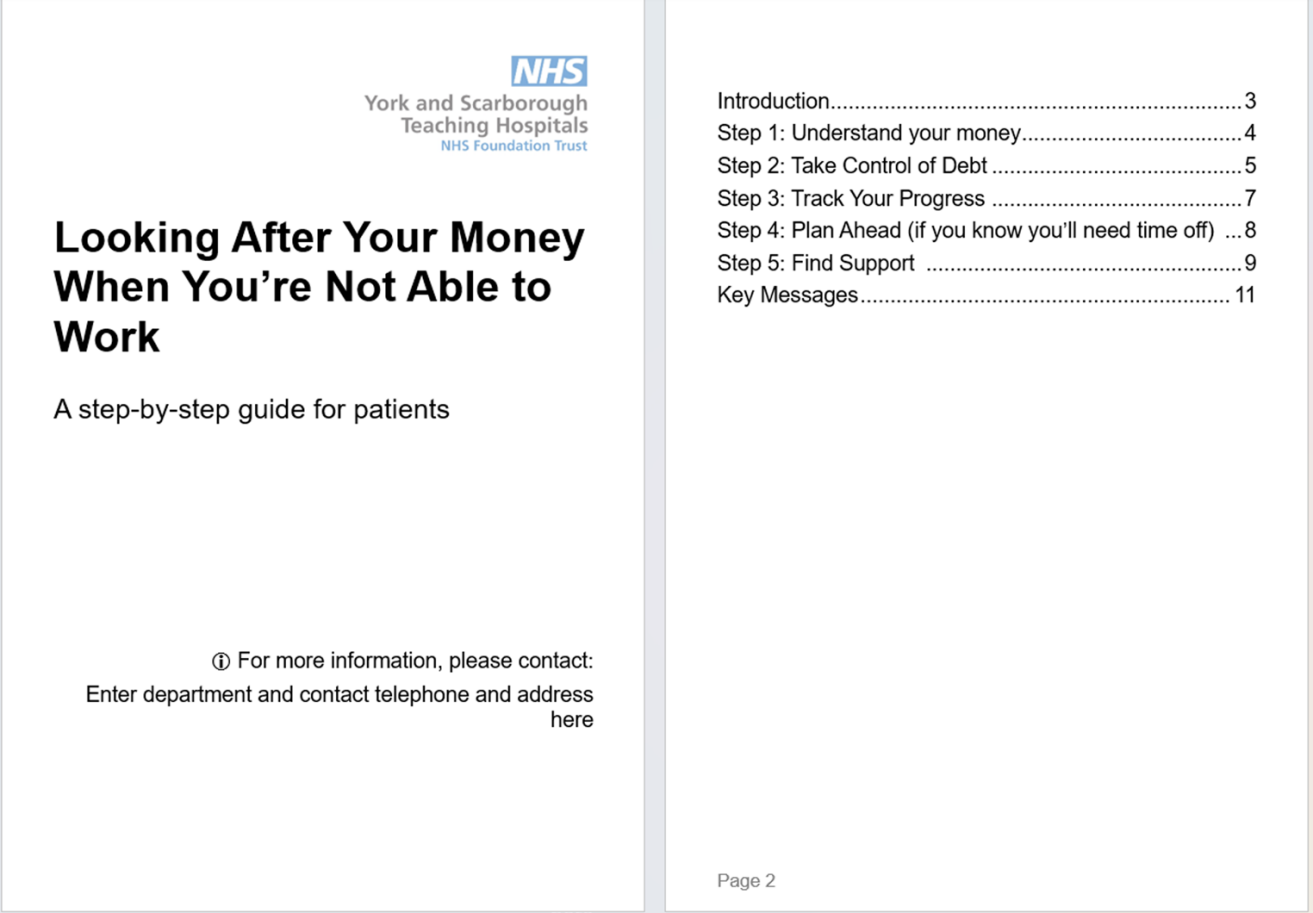
Patient information leaflet on “looking after your money when you’re not able to work”
- Step 2: *“Guided Support and Advocacy”* might involve facilitated group discussions, workshops on financial planning, or signposting to occupational health services, welfare rights advice, or peer support—particularly for those facing job insecurity or career transitions.
- Step 3: *“Specialist Employment and Financial Intervention”* would include personalised input from vocational rehabilitation teams, benefits advisors, or clinical psychologists with expertise in health-related occupational disruption. These services would be most appropriate for individuals experiencing significant financial hardship, sustained work absence, or associated psychological distress. This stepped care approach, shown in Fig. 3, acknowledges the varying degrees of employment-related impact in CAWH and aims to provide tailored, graduated support responsive to patient need.

To implement changes, we propose a stepped care model to guide support for individuals living with CAWH [22]:

- Step 1: *“Awareness and Understanding”* could include the development of a patient-facing information leaflet on “AWH and Work.” Such a resource, written in Plain English and grounded in real experiences, could help normalise common employment-related concerns, provide practical guidance on navigating workplace conversations, and offer information on rights at work, sick pay entitlements, and vocational adaptations. To support this, we have developed a patient information leaflet summarising financial considerations for patients with chronic illness, irrespective of operative or non-operative intervention, with specific advice on budgeting, debt management, and sources of local and national support. This resource has been designed for provision in outpatient clinics and is available as Fig. 2 and in the Supplementary File X.

**Fig. 3:**
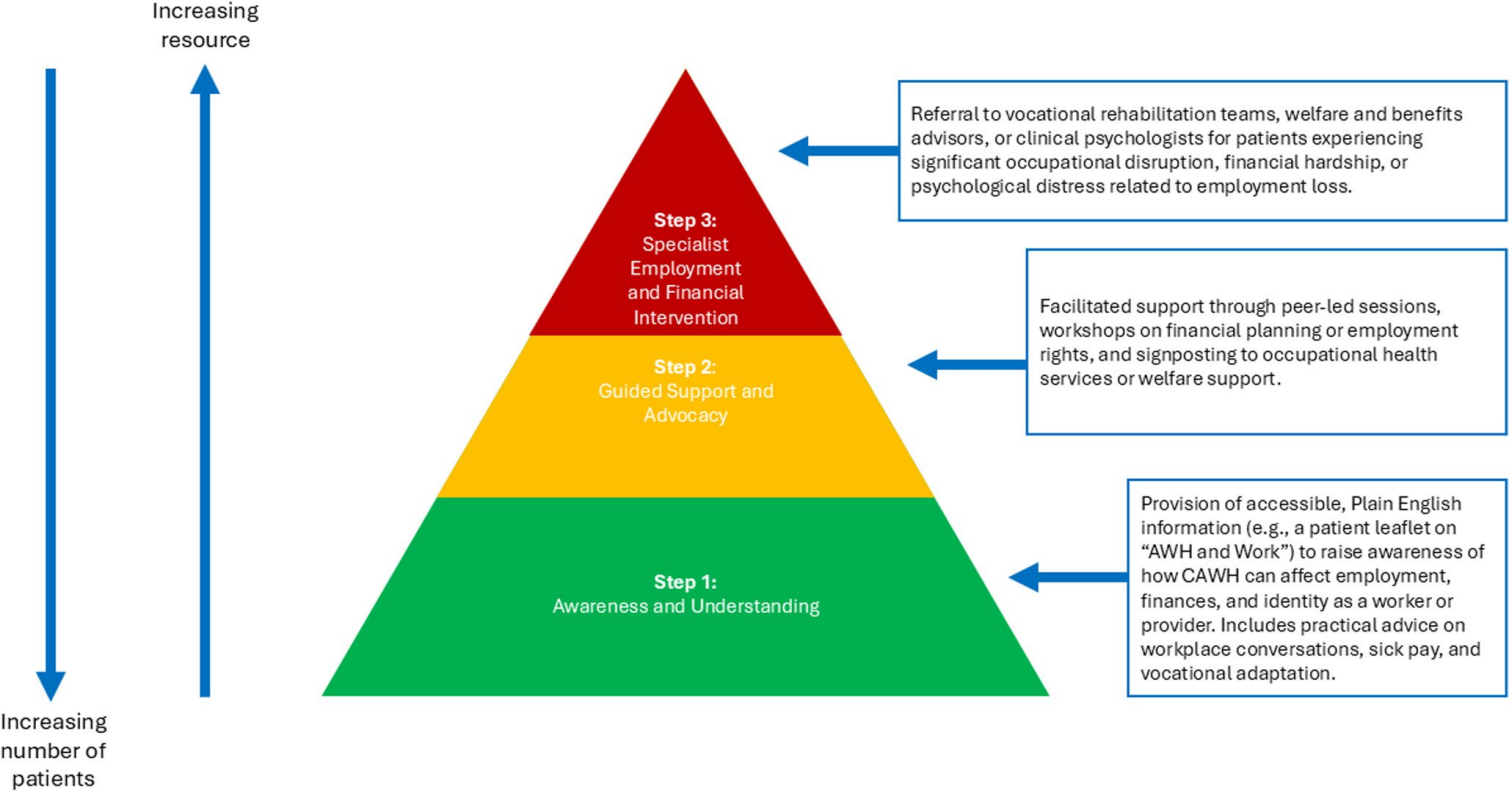
Stepped care model for employment and finance support in AWH

This study sought to explore the challenges that patients with CAWH experience in relation to employment, with the hope that this may lead to meaningful ways of supporting patients through this difficulty. Using a qualitative methodology grounded in a phenomenological approach, we were able to capture the nuanced, lived experiences of patients affected by CAWH. While this approach offers rich, in-depth insights, future research may benefit from quantifying both the direct and indirect economic impacts of CAWH on patients and their families or care networks, providing a more comprehensive understanding of the burden of this condition. Quantifying these costs could help inform clinical and policy decision-making by identifying areas where interventions might be most cost-effective, and by better capturing the broader socioeconomic impact of disease on individuals and households [20].

We recognise that the implementation of educational, vocational or psychological programmes may vary considerably across healthcare systems, and resource constraints may limit the extent to which stepped support can be delivered uniformly. The model proposed here is intended as a conceptual framework that can be adapted to local capacity, workforce availability and service configuration, rather than a prescriptive set of interventions.

### Study limitations

This study employed a phenomenological approach, prioritising rich, first-person narratives to explore how individuals experience and interpret the symptoms and restrictions imposed by AWH. This method relies on participants’ ability to reflect upon, remember, and articulate their experiences. As such, it may miss forms of knowledge that are enacted through the body but go unspoken (for example, subtle compensatory movements or unconscious micro-adjustments). These physical strategies are often habitual rather than narrated and may therefore remain invisible in interview-based data collection.

Our participants were drawn from a single, highly-specialised tertiary abdominal wall unit in the UK. Efforts were made, through purposive sampling, to ensure demographic diversity across age, gender, and VWHG grade. Participants were recruited from a specialist abdominal wall clinic and may differ from individuals who do not seek or have access to tertiary care. However, the demographic and clinical characteristics of the sample reflect those commonly seen in CAWH pathways within UK specialist centres. However, the views of individuals who cannot access specialist care, or are managed entirely in primary care may differ substantially and warrant further exploration. All interviews were conducted by a single researcher with a clinical background and prior familiarity with abdominal wall hernia care. While this shared knowledge may have facilitated rapport, it may also have introduced implicit biases. Also, the heterogeneity of participants’ age, employment status and comorbidities may influence how financial or occupational consequences were experienced. While purposive sampling was intentional to reflect the diversity of real-world CAWH populations, we acknowledge that this may limit transferability of specific thematic nuances. Participants may have shaped their narratives based on assumptions about what the interviewer already knew, or what they felt was relevant or appropriate to share in a medical context. To mitigate this, the interviewer engaged in ongoing reflexivity throughout the study. Data collection occurred during the early COVID-19 pandemic, a period associated with heightened employment uncertainty. While participants were asked to reflect on hernia-related impacts specifically, we recognise that wider socioeconomic conditions may have shaped how some experiences were articulated.

The role of the interviewer (OS) also warrants consideration. OS is a cis-presenting, Caucasian, young, female doctor with surgical and research training, as well as operative experience in CAWH. She had no prior relationship with participants before the interviews. Nonetheless, OS engaged in training on cognitive interviewing prior to interviews, and active reflexivity throughout the study, recognising that her demographic and professional background may have shaped how participants related to her, and potentially influenced how the data were interpreted. This reflexive stance was supported by regular analytic discussions with the broader research team, including those outside of surgical practice, to reduce bias and support balanced interpretation.

## Conclusion

This study found that CAWH resulted in job loss, the financial burden of this, as well as a negative psychological impact in these participants. Improved pre-operative information and holistic bio-psycho-emotional support may improve the patient experience and help both patients and surgeons prepare for realistic results regarding the financial impact of CAWH. We have paved the way for a stepped care model, offering scalable support from general awareness to specialist intervention, which may provide a practical framework for addressing the diverse concatenation of employment and financial challenges, amongst wider quality of life impacts, faced by this patient group.

## Supplementary Information

Below is the link to the electronic supplementary material.

Supplementary Material 1 (DOCX 28.9 KB)

Supplementary Material 2 (DOCX 26.5 KB)

Supplementary Material 3 (DOCX 622 KB)

Supplementary Material 4 (DOCX 176 KB)
