# Supplementary figures and images for: Novel insights into abdominal wall hernia (AWH) and its negative impact on patients’ finances: “Doing my job was pretty impossible”
Source: Hernia. 2026 Feb 4;30(1):85. doi: 10.1007/s10029-026-03596-9 (PMC12872652; doi:10.1007/s10029-026-03596-9)

**Supplementary File 3:** interview schedule


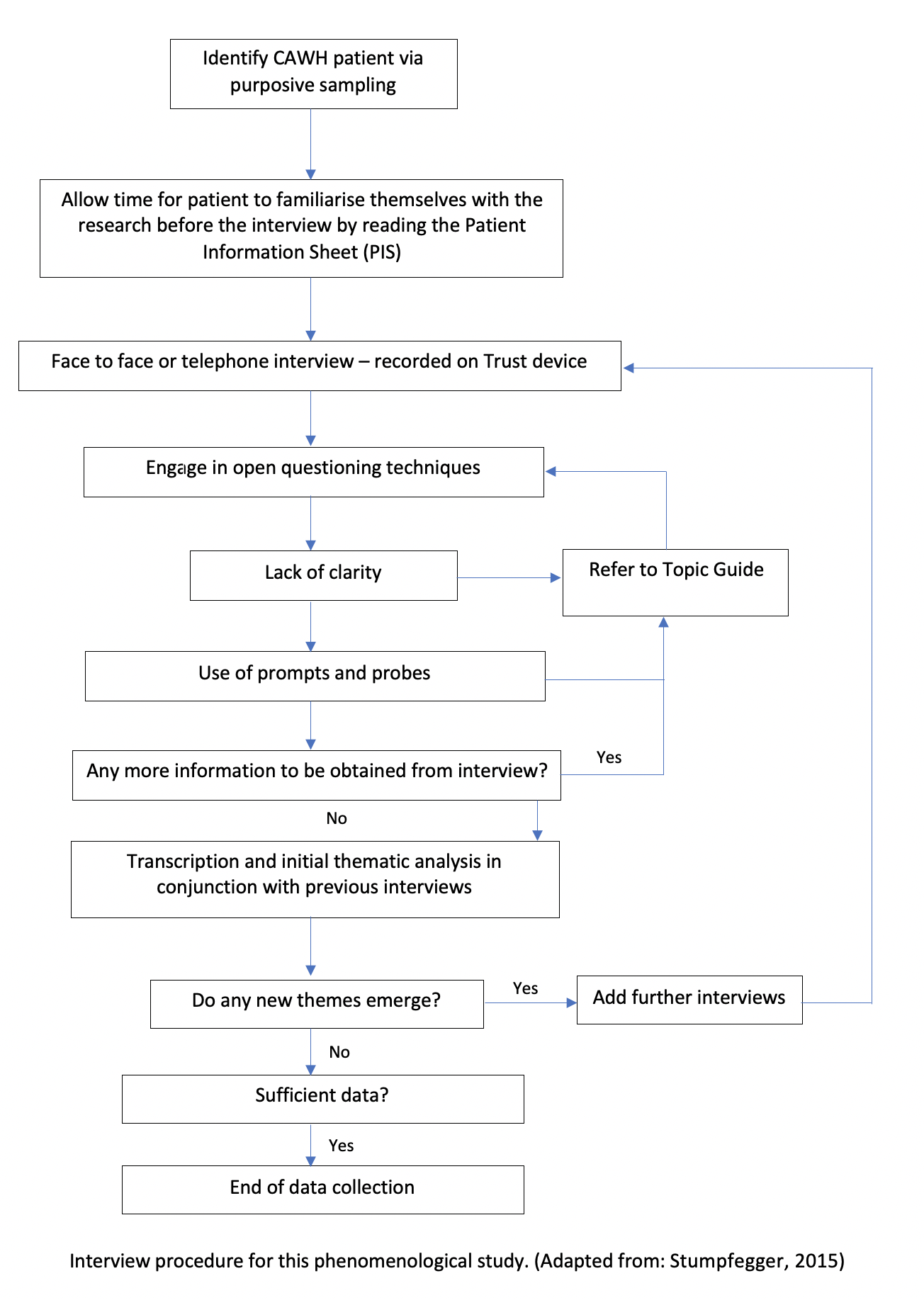

Supplement: Supplementary file 3 — Supplementary Material 3 (DOCX 622 KB) [file 10029_2026_3596_MOESM3_ESM.docx]
