# Supplementary material for: Novel insights into abdominal wall hernia (AWH) and its negative impact on patients’ finances: “Doing my job was pretty impossible”
Source: Hernia. 2026 Feb 4;30(1):85. doi: 10.1007/s10029-026-03596-9 (PMC12872652; doi:10.1007/s10029-026-03596-9)
Supplement: Supplementary file 4 — Supplementary Material 4 (DOCX 176 KB) [file 10029_2026_3596_MOESM4_ESM.docx]

**Looking After Your Money When You’re Not Able to Work**

A step-by-step guide for patients

🛈 For more information, please contact:

Enter department and contact telephone and address here

[Introduction](#_Toc178003278) 3

Step 1: Understand your money [4](#_Toc178003279)

Step 2: Take Control of Debt [5](#_Toc178003280)

Step 3: Track Your Progress  [7](#_Toc178003281)

Step 4: Plan Ahead (if you know you’ll need time off) 8

Step 5: Find Support  [9](#_Toc178003283)

Key Messages [11](#_Toc178003284)

|  |  |
| --- | --- |
|  |  |

Introduction

Being unwell or having surgery can sometimes mean taking time off work. This may affect your income and can cause extra stress. Worrying about money is normal, but there are practical steps you can take. This guide will help you manage your finances and show you where to get support.

This leaflet will help you:

- Understand your finances clearly
- Make practical choices about spending
- Get support for managing debt
- Find out what financial help is available
- Reduce stress by taking small steps towards control

You don’t need to do everything at once. Start with one step, and move forward at your own pace.

Step 1: Understand your money

When money feels tight, the first and most important step is to get a clear picture of your finances.

- **Make a monthly budget**. Write down all the money you have coming in (such as wages, benefits, pensions, or savings) and all the money going out (such as rent or mortgage, food, household bills, travel, subscriptions, and leisure).
- **Check the facts**. Look at your last three months of bank statements to make sure your list is realistic and accurate. It is easy to forget small regular costs (like coffees, streaming services, or delivery charges) and they can quickly add up.
- **Sort your spending into categories**. Mark which payments are essential (things you cannot avoid, like housing, energy, food) and which are non-essential (things you could cut back on, such as takeaways, subscriptions, or other discretionary items).

Having everything written down helps you see where your money is going. Even small changes can make a difference.

There are free online tools and budgeting apps (such as MoneyHelper or Citizens Advice budget planner) that make this easier.

Step 2: Take Control of Debt

Debt can quickly add to stress when you are not working, but there are ways to manage it.

- Make a full list. Write down all debts, including credit cards, loans, overdrafts, store cards, or “buy now, pay later” agreements. For each one, note the interest rate, the monthly repayment, and how long it will take to clear.
- Pay the right ones first. Some debts are more urgent than others. Missing rent, council tax, or utility bills can have serious consequences. Credit cards and loans can also grow quickly if interest is high. Start by focusing on these, while making minimum payments on others.
- Talk to creditors early. If you cannot make a payment, contact your bank, lender, or utility provider straight away. Most companies have hardship policies and can offer reduced payments, freezes on interest, or payment holidays.
- Get professional advice. Independent charities and advice services can help you make a debt plan. You do not need to pay for debt advice – free support is available (see “Where to Get Help” below).
- Avoid more borrowing. Taking on new loans to cover old ones can make things worse. If consolidation is right for you, it should only be done with professional advice.

Remember: you are not alone. Many people face debt during illness, and support services are experienced in helping people through it.

**Step 3: Track Your Progress**

Making a plan is only the beginning.

Illness and recovery can be unpredictable, so it’s normal for your finances to change. This step is about checking in regularly and adjusting when needed.

- Review each month. Sit down once a month to check your spending against your budget.
- Be flexible. Health issues can mean sudden changes. If your situation changes, adjust your plan rather than feeling you have “failed.”
- Use envelopes or apps. Some people find it easier to split money into weekly “pots” (either physically with envelopes or digitally with banking apps). This helps avoid overspending early in the month.
- Notice the positives. Celebrate small wins, such as reducing a bill or paying off part of a debt. Every step forward reduces stress and builds confidence. Keep a simple list or tick sheet – for example, “credit card balance reduced by £50 this month” – so you can see how far you’ve come.
- Involve family if you can. Talking openly about money with those close to you can reduce pressure and help share responsibility.

**Step 4: Plan Ahead (if you know you’ll need time off)**

If your surgery or treatment is planned in advance, you can prepare your finances early.

- **Save a little if possible**. Even a small emergency fund can help with unexpected costs.
- **Pay down high-interest debts**. Reducing these early can prevent them from growing while you are off work.
- **Practice living on less**. Try spending only what you expect to have during recovery for a few weeks. This shows where adjustments are needed.
- **Talk to your employer if you are working.** Ask about sick pay, benefits, or flexible working options. Your HR department may also know about financial support schemes.
- **Think short-term**. These changes are often temporary and linked to your recovery period.

Step 5: Find Support

You may be entitled to extra financial support while you are unable to work.

**Government Support:**

- Universal Credit
  - support for people on low income or out of work
- Options for dealing with debt
  - practical advice for managing debt
- Benefits advice
  - full list of benefits you may qualify for

**Independent Advice:**

- Citizens Advice
  - free advice on benefits, debt, and rights
- MoneyHelper
  - free government-backed money and pensions guidance
- MoneySavingExpert Benefits Calculator
  - check what benefits you may be entitled to

Don’t wait until money problems get worse. Free help is available, and seeking advice early makes things easier to manage.

Key Messages

✔ Write a budget so you know where you stand.

✔ Be realistic and honest about what is essential.

✔ Don’t ignore debt – take action early and get support.

✔ Ask about benefits and entitlements.

✔ Remember: your financial challenges are often temporary. Taking small, steady steps can make a big difference.

Tell us what you think of this leaflet

We hope that you found this leaflet helpful. If you would like to tell us what you think, please contact:
[enter contact details here including address and telephone and email if possible.]

Teaching, training and research

Our Trust is committed to teaching, training and research to support the development of health and healthcare in our community. Healthcare students may observe consultations for this purpose. You can opt out if you do not want students to observe. We may also ask you if you would like to be involved in our research.

Patient Advice and Liaison Service (PALS)

PALS offers impartial advice and assistance to patients, their relatives, friends and carers. We can listen to feedback (positive or negative), answer questions and help resolve any concerns about Trust services.

PALS can be contacted on 01904 726262, or email
[yhs-tr.patientexperienceteam@nhs.net](mailto:yhs-tr.patientexperienceteam@nhs.net).

An answer phone is available out of hours.

Leaflets in alternative languages or formats

If you would like this information in a different format, including braille or easy read, or translated into a different language, please speak to a member of staff in the ward or department providing your care.

Patient Information Leaflets can be accessed via the Trust’s Patient Information Leaflet website: [www.yorkhospitals.nhs.uk/your-visit/patient-information-leaflets/](http://www.yorkhospitals.nhs.uk/your-visit/patient-information-leaflets/)

| Owner | Andrew Bertram |
| --- | --- |
| Date first issued | Enter date v1 issued |
| Review Date | Enter review date (no more than three years from issue) |
| Version | x (issued XXX 2023) |
| Approved by | Enter approval group here |
| Document Reference | PILxxx vx |

© 2024 York and Scarborough Teaching Hospitals NHS Foundation Trust.

All Rights reserved.
